# Activation of mTOR mediates hyperglycemia-induced renal glomerular endothelial hyperpermeability via the RhoA/ROCK/pMLC signaling pathway

**DOI:** 10.1186/s13098-021-00723-7

**Published:** 2021-10-09

**Authors:** Xiaolin Chen, Jianhui Chen, Xianfan Li, Zengpu Yu

**Affiliations:** 1 Department of Clinical Laboratory, Pingxiang People’s Hospital, Pingxiang, 337000 Jiangxi China; 2 Department of Clinical Laboratory, The Sixth Clinical College of Gannan Medical University, Pingxiang, Jiangxi China

**Keywords:** Glomerular endothelial cell, Permeability, Myosin light chain, High glucose, RhoA/ROCK

## Abstract

**Objective:**

Hyperglycemia is associated with albuminuria and renal glomerular endothelial dysfunction in patients with diabetic nephropathy. The mTOR and RhoA/ROCK signaling pathways are involved in glomerular filtration barrier (GFB) regulation, but their role in high glucose (HG)-induced GFB dysfunction in human renal glomerular endothelial cells (HRGECs) has not been investigated. This study aimed to investigate the mechanisms of HG-induced GFB dysfunction in vitro.

**Materials and methods:**

HRGECs were cultured in vitro and exposed to HG. The horseradish peroxidase–albumin leakage and transendothelial electrical resistance of the endothelial monolayer were measured after HG treatment with or without rapamycin preincubation. A fluorescence probe was used to study the distribution of F-actin reorganization. The phosphorylation levels of myosin light chain (MLC) and mTOR were measured via western blotting. RhoA activity was evaluated via GTPase activation assay. The effects of blocking mTOR or the RhoA/ROCK pathway on endothelial permeability and MLC phosphorylation under HG conditions were observed.

**Results:**

HG exposure induced F-actin reorganization and increased MLC phosphorylation, leading to EC barrier disruption. This effect was attenuated by treatment with rapamycin or Y-27632. Phospho-MLC (pMLC) activation in HRGECs was mediated by RhoA/ROCK signaling. mTOR and RhoA/ROCK inhibition or knockdown attenuated pMLC activation, F-actin reorganization and barrier disruption that occurred in response to HG exposure.

**Conclusions:**

Our results revealed that HG stimulation upregulated RhoA expression and activity through an mTOR-dependent pathway, leading to MLC-mediated endothelial cell cytoskeleton rearrangement and glomerular endothelial barrier dysfunction.

## Introduction

Chronic hyperglycemia is the leading cause of diabetic nephropathy [1], which is characterized by albuminuria in the early stage [2, 3]. The pathogenesis of albuminuria in diabetes is complex and involves glomerular filtration barrier (GFB) dysfunction [3–5]. Human renal glomerular endothelial cells (HRGECs) interact with podocytes, contributing to the integrity of the GFB [3, 6, 7]. Hyperglycemia increases GFB permeability in vivo in rats and mice [8], and previous studies have shown that high glucose (HG)-induced GFB disruption is associated with the development and progression of GFB hyperfiltration [5, 9, 10]. Furthermore, a recent investigation showed that HG exposure leads to hyperpermeability and increased albumin leakage in both renal [11] and non-renal endothelial cells [12], which could be linked to F-actin–dependent cytoskeleton rearrangement.

Changes in endothelial contractility lead to increased permeability, which is affected by the activity of actin and myosin [3, 7, 13]. Activation of the Rho A GTPases and the downstream Rho kinase (ROCK), induce myosin light chain (MLC) phosphorylation [12, 14], which plays a major role in maintaining endothelial cell barrier integrity [15]. MLC phosphorylation facilitates contraction of the F-actin cytoskeleton and the creation of intercellular gaps between endothelial cells, thereby increasing GFB permeability [7, 9, 13, 16]. RhoA/ROCK activation has been shown to be involved in the glomerular hyperfiltration of albumin and microvascular/retinal complications of hyperglycemia [11, 17].

Mechanistic target of rapamycin (mTOR), a protein kinase [18], is a cytosolic enzyme associated with cellular growth and homeostasis via mTOR complexes 1 and 2. Several lines of evidence suggest that the mTOR pathway may directly regulate GFB function [19, 20], which is a key player in diabetic kidney disease. Accumulating evidence suggests that mTOR activation can lead to GFB dysfunction via various signaling pathways under HG conditions [19, 21]. Additionally, blockade of mTOR activation can suppress the progression of diabetic kidney disease, primarily by reducing glomerular hyperpermeability and mitigating proteinuria [20]. The underlying mechanisms of HG-induced aberrant glomerular filtration and the effects of mTOR on changes in the GFB are presently unknown. mTOR controls the expression of a variety of downstream target proteins, including the ribosomal protein S6 kinase (S6K) [18]. Liu et al. demonstrated that mTOR is involved in the expression and activation of RhoA that mediates F-actin reorganization and cell motility [22]. Therefore, we hypothesized that mTOR-mediated protein synthesis and RhoA activity lead to increased F-actin reorganization and endothelial cell hyperpermeability under hyperglycemic conditions. We also examined the roles of MLC and the RhoA/ROCK signaling pathway in HG-induced endothelial hyperpermeability.

## Materials and methods

### Chemicals and reagents

Primary antibodies against phospho-MLC (3674), MLC (8505), phospho-mTOR (2971), mTOR (2983), α-tubulin (3873), GAPDH (2118), phospho-RPS6 (4585), and RPS6 (2217); the RhoA Activation Assay Kit (8789); and rapamycin (8789) were purchased from Cell Signaling Technology (Danvers, MA, USA). FITC-Phalloidin (P5282) and Y-27632 (S1049) were purchased from Sigma Aldrich (Saint Louis, MO, USA) and Med Chem Express (Newark, NJ, USA), respectively.

### Cell culture and glucose treatment

HRGECs (PS-4000) were provided by ScienCell Research Laboratories (Kirkland, WA, USA). HRGECs were maintained in endothelial cell medium with 1% endothelial cell growth supplement and 10% serum (ScienCell Research Laboratories). HRGECs were pretreated with or without rapamycin for 2 h and then stimulated with either 5.5 mM glucose (normal glucose; NG) or 30 mM glucose (high glucose; HG) (R&D Systems) for 16 h before western blotting or immunofluorescence analysis.

### Western blot analysis

Western blotting was performed as described previously [12]. Briefly, HRGECs were lysed with lysis buffer containing proteinase and phosphatase inhibitors (Roche, USA). Protein concentration was measured via BCA assay (Beyotime, Beijing, China). The same amount of protein was isolated using SDS-PAGE and then transferred to PVDF membranes. The membranes were blocked with 5% non-fat milk and incubated with primary antibody overnight at 4 °C. Then, the membranes were probed with horseradish peroxidase (HRP)-conjugated secondary antibody for 2 h at 20–24 ℃. Protein bands were detected using ECL Super Signal reagent (Pierce, 34078) and visualized using a digital gel image analysis system (BIO RAD, Hercules, CA, USA).

### F-actin staining assay

For the F-actin stress fiber immunofluorescence assay, confluent HRGEC monolayers were grown on glass coverslips precoated with 0.1% gelatin. After treatment, the cells were fixed with 4% paraformaldehyde for 10 min and then blocked with PBS containing 1% bovine serum albumin. Next, the cells were incubated with FITC-Phalloidin for 1 h and stained with DAPI. Confocal images were acquired with a laser-scanning confocal microscope (FV1000-IX81, Olympus). Image analysis was performed using FV10-ASW Viewer software (Ver 4.1, Olympus Life Science, Japan).

### Transendothelial electrical resistance (TEER) assay

HRGECs were seeded on transwell inserts (0.4 µm pore, Millipore, USA) and grown to confluence. The TEER of the HRGEC monolayer was measured using a Millicell-ERS voltohmmeter (Millipore, Burlington, MA, USA). Resistance values of the experimental groups are shown in units of Ω cm^2^. The transwell TEER of each group was recorded and normalized by subtracting the baseline TEER.

### Transendothelial albumin permeability assay

HRGECs were grown to confluence on a 0.4 μm pore transwell insert (3413, Coring). After treated with NG or HG with or without inhibitors for 24 h, medium containing horseradish peroxidase (HRP)-labeled albumin (50 μg/mL; Solarbio, Beijing, China) was added to the top chamber. The concentration of albumin in the chambers was measured using the TMB Soluble Substrate kit (Solarbio, China), and absorbance values were recorded using a microplate reader (Elx 800, BioTek). The permeability coefficient of albumin (Pa) was calculated using the equation Pa = [A]/*t* × 1/*A* × V/[L], where [A] and [L] represent the albumin concentration in the bottom and top chambers, respectively; *t* represents time (s), *A* represents the area of the membrane (cm^2^), and V represents the volume of the bottom chamber (uL).

### Rho activity assay

Rho activity in HRGECs was analyzed using the Active Rho Detection Kit. Briefly, HRGECs grown in 100 mm petri dishes were treated with either 5.5 or 30 mM glucose and/or pretreated with rapamycin (100 nM, 1 h). Next, the cells were lysed with cell lysis buffer and incubated with rhotekin Rho-binding peptide (GST-Rhotekin-RBD) immobilized on agarose to pull down GTP-bound Rho. The expression of activated GTP-Rho and total RhoA was detected using western blotting.

### Cell transfection

siRNA against RhoA (si-RhoA, sc-29471) and a negative control (si-control, sc-37007) were provided by Santa Cruz Biotechnology (Dallas, Texas, USA). The cells were transfected with the indicated siRNA using a 4D-Nucleofector system (Lonza, Alpharetta, GA, USA) according to the manufacturer’s protocol. After 48 to 72 h, cells were treated with NG or HG and then harvested and analyzed.

### Statistical analysis

Statistical analyses were performed using GraphPad Prism 7.0 software (La Jolla, CA, USA). Data are presented as the mean ± standard error. One-way ANOVA with the Newman–Keuls test for post hoc comparisons was performed to test for differences among multiple groups. Student’s *t*-test was used for comparisons between two groups. Values of P ≤ 0.05 were considered significant.

## Results

### HG activated the mTOR signaling pathway in HRGECs

**Fig. 1:**
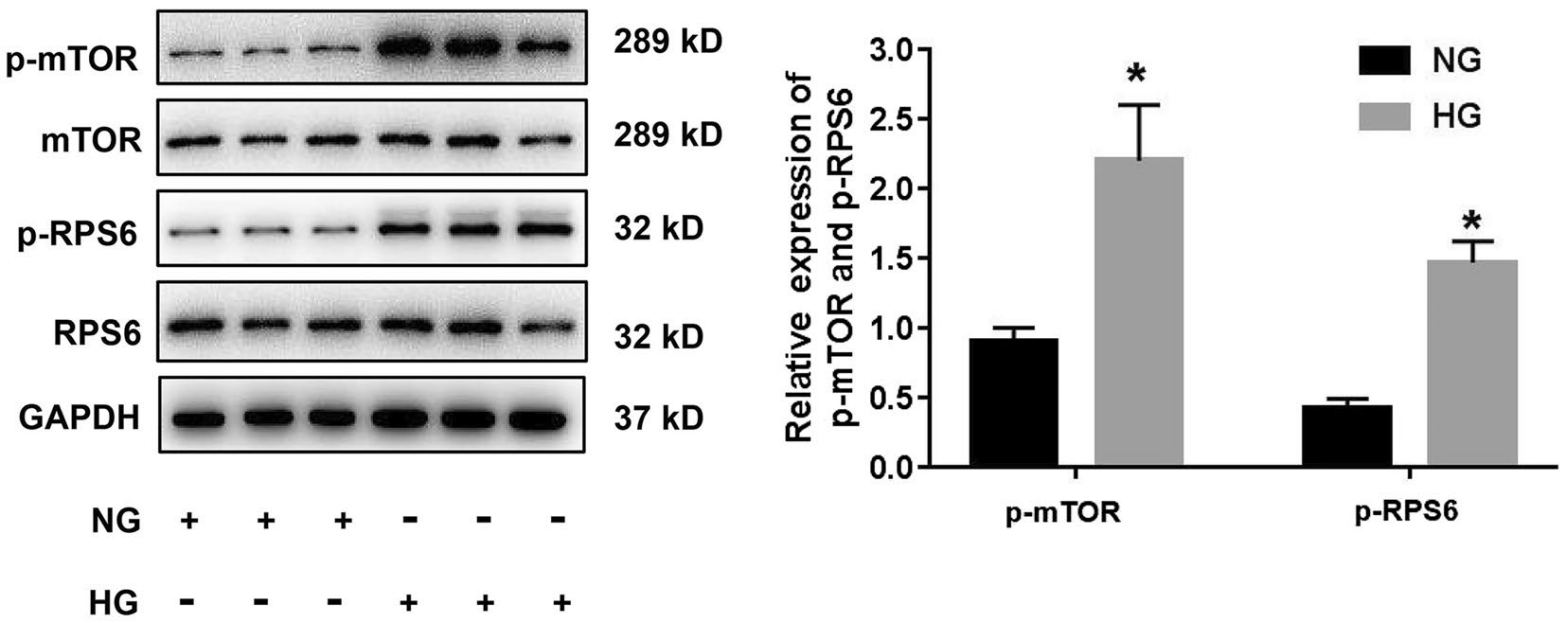
HG activated the mTOR pathway in HRGECs. HRGECs were cultured in vitro and treated with 5.5 mM glucose (NG) or 30 mM glucose (HG) for 24 h. Representative blots indicate the expression of mTOR and RPS6 and the levels of p-MTOR and p-RPS6. HG, high glucose; HRGECs, human renal glomerular endothelial cells; NG, normal glucose. * indicates p < 0.05 vs. the NG group

To examine mTOR pathway activation, HRGECs were co-cultured with NG or HG for 24 h. The phosphorylation of mTOR and RPS6 was significantly increased under HG conditions compared to that under NG conditions (p < 0.05) (Fig. 1).

### Inhibition of mTOR reduced HG-induced hyperpermeability in HRGECs

**Fig. 2:**
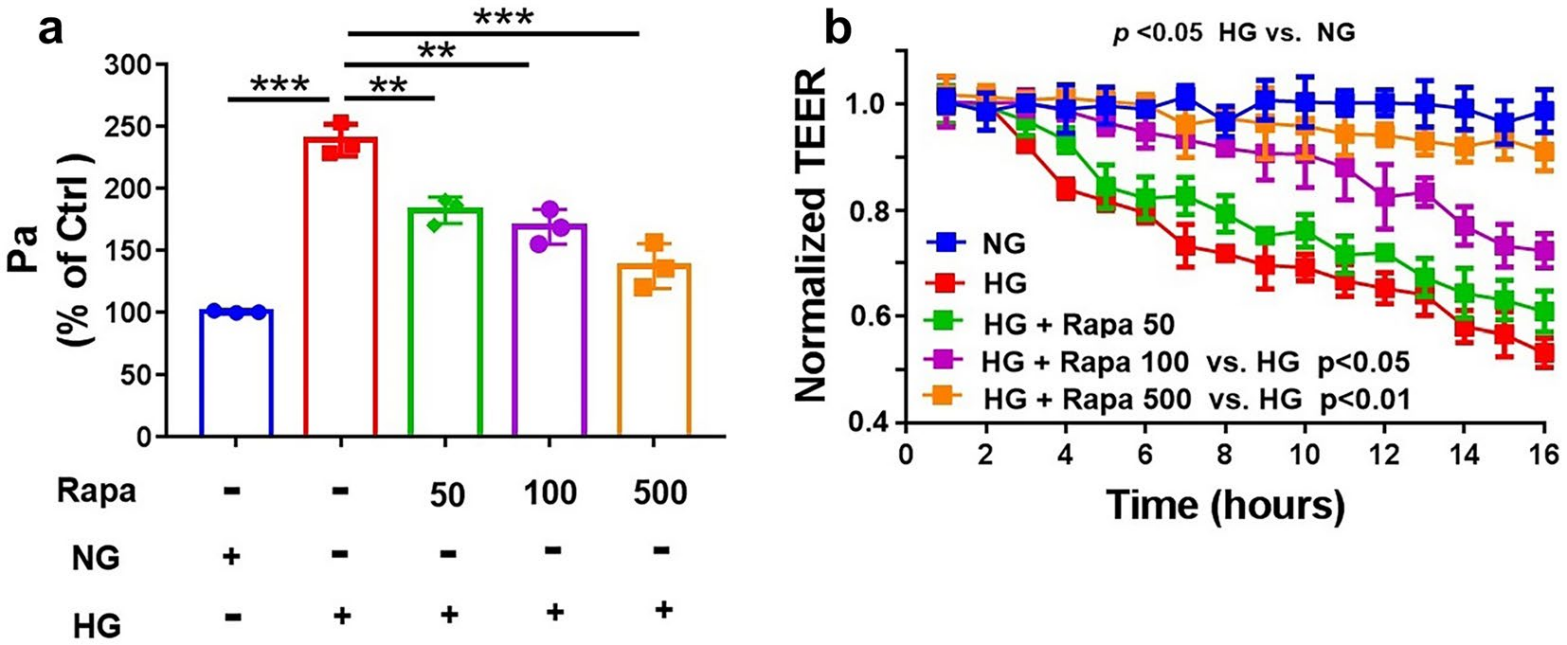
Rapamycin reversed hyperpermeability in HG-cultured HRGECs. The TEER (**a**) and HRP-albumin permeability (Pa) of transendothelial flux (**b**) were measured in HRGECs treated with rapamycin (0, 50, 100, or 500 nM) for 2 h with or without HG (30 mM) stimulation for 22 h. TEER was normalized to the baseline before treatment (TEER_0_) (n = 3). HG, high glucose; HRGECs, human renal glomerular endothelial cells; TEER, transendothelial electric resistance; HRP, horseradish peroxidase. ** indicates p < 0.01; *** indicates p < 0.001

The permeability of cultured endothelial cell monolayers can be significantly increased by HG stimulation [12]. To examine the effect of mTOR inhibition on HG-induced endothelial barrier dysfunction, we pretreated HRGECs either with HG alone or with HG plus 0, 50, 100, or 500 mM rapamycin for 24 h. HRP-albumin leakage through the HRGEC monolayer was enhanced in the HG group compared with that in the NG group (*p* < 0.05) (Fig. 2a). However, the inhibition of mTOR by rapamycin inhibited HG-induced endothelial hyperpermeability in a dose-dependent manner.

In addition, the TEER of endothelial monolayers serves as a strong indicator of the integrity of cellular barriers. HG conditions profoundly decreased the TEER of the cells (*p* < 0.05) (Fig. 2b), and rapamycin abolished this effect, with the TEER slowly returning to baseline levels.

### HG-induced endothelial cell barrier dysfunction correlated with F-actin cytoskeleton changes and MLC phosphorylation

**Fig. 3:**
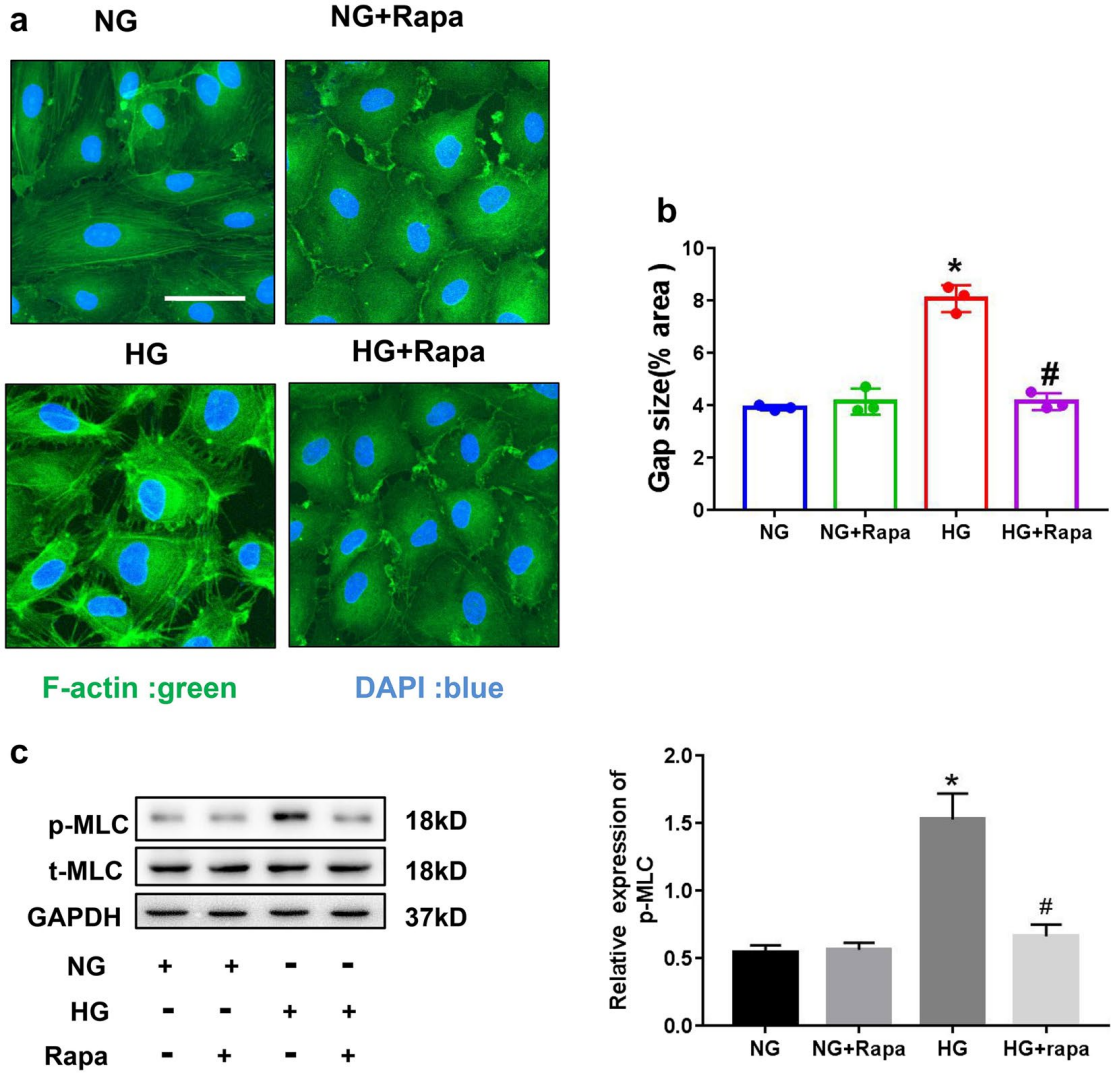
Rapamycin suppressed HG-induced barrier dysfunction by downregulating MLC phosphorylation. **a** Rapamycin (100 nM) pretreatment for 2 h, HRGECs were incubated with NG or HG for 22 h. F-actin stress fiber formation was assessed via FITC-Phalloidin staining. Cells were stained for F-actin (green) and DAPI (blue) and then imaged. **b** The intercellular gap area in each group was measured; n = 4. **c** The levels of MLC phosphorylation were analyzed using western blotting. HG, high glucose; MLC, myosin light chain; HRGECs; human renal glomerular endothelial cells; NG; normal glucose. * indicates p < 0.05 vs. the NG group. # indicates p < 0.05 vs. the HG group

MLC phosphorylation is closely linked to F-actin rearrangement, which leads to endothelial cell barrier disruption via intercellular gap formation [23]. As shown in Fig. 3a, HG conditions induced the formation of actin stress fibers and the appearance of gaps at cell–cell junctions (*p* < 0.05). Rapamycin blocked the formation of actin stress fibers and decreased the number of intracellular gaps induced by HG conditions (Fig. 3b).

To examine the mechanisms of the HG-induced formation of actin stress fibers, MLC phosphorylation was analyzed using western blotting. HG treatment of HRGECs produced an increase in MLC phosphorylation levels that was attenuated by rapamycin (Fig. 3c). These data suggested that mTOR activation is involved in the capacity of HG to induce a key signaling cascade in endothelial cells, leading to MLC phosphorylation and actin stress fiber formation.

### RhoA/ROCK signaling pathway was required for HG-induced renal endothelial barrier dysfunction

**Fig. 4:**
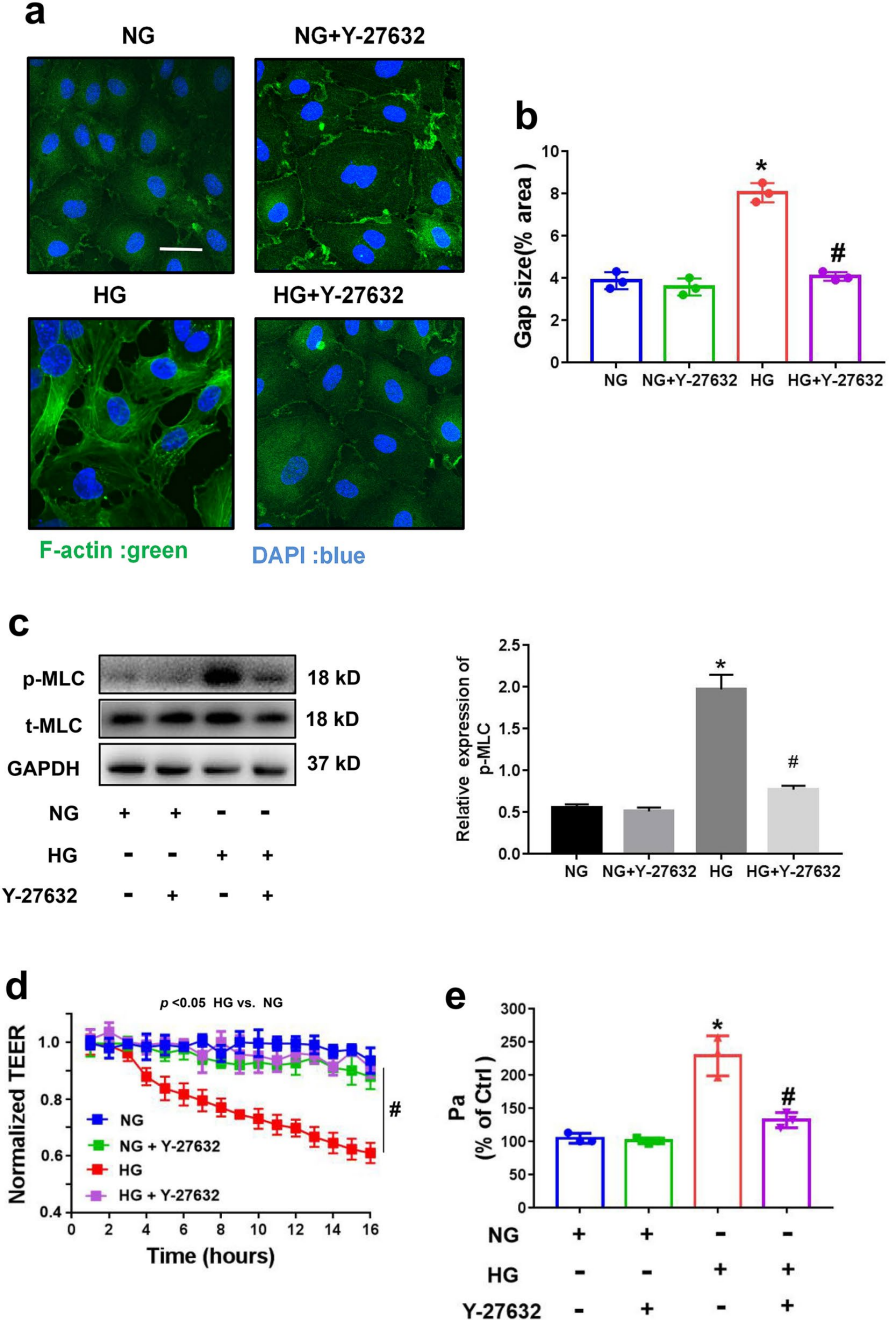
HG induced the formation of stress fibers and intercellular gaps in a RhoA/ROCK pathway–dependent manner. **a** HRGECs were incubated with 5.5 mM glucose (NG) or 30 mM glucose (HG) and/or the ROCK inhibitor Y-27632 (10 μM) for 24 h. F-actin stress fiber formation was assessed using FITC-phalloidin staining. Cells were stained for F-actin (green) and DAPI (blue) and then imaged. **b** The intercellular gap area in each group (n = 3). **c** Western blot analysis of MLC phosphorylation.** d**–**e** Preincubation with Y-27632 (10 μM) significantly reduced the HG-induced increase in permeability according to TEER measurements and the HRP-albumin leakage assay (n = 3). HG, high glucose; HRGECs, human renal glomerular endothelial cells; NG, normal glucose; MLC, myosin light chain; TEER, transendothelial electrical resistance; HRP, horseradish peroxidase. * indicates p < 0.05 vs. the NG group. # indicates p < 0.05 vs. the HG group

Upstream RhoA/ROCK signaling drives MLC phosphorylation and intercellular gap formation [14, 23]. We explored whether the RhoA/ROCK pathway participates in HG-induced endothelial cell hyperpermeability. HRGEC monolayers were preincubated with HG and the ROCK inhibitor Y-27632. Y-27632 abolished HG-induced actin stress fiber formation (Fig. 4a–b), MLC phosphorylation (Fig. 4c), and permeability increase (*p* < 0.05) (Fig. 4d–e). These results indicated that the RhoA/ROCK pathway was critical for establishing HG-induced hyperpermeability in renal endothelial cells.

### HG increased RhoA activation and expression in HRGECs

**Fig. 5:**
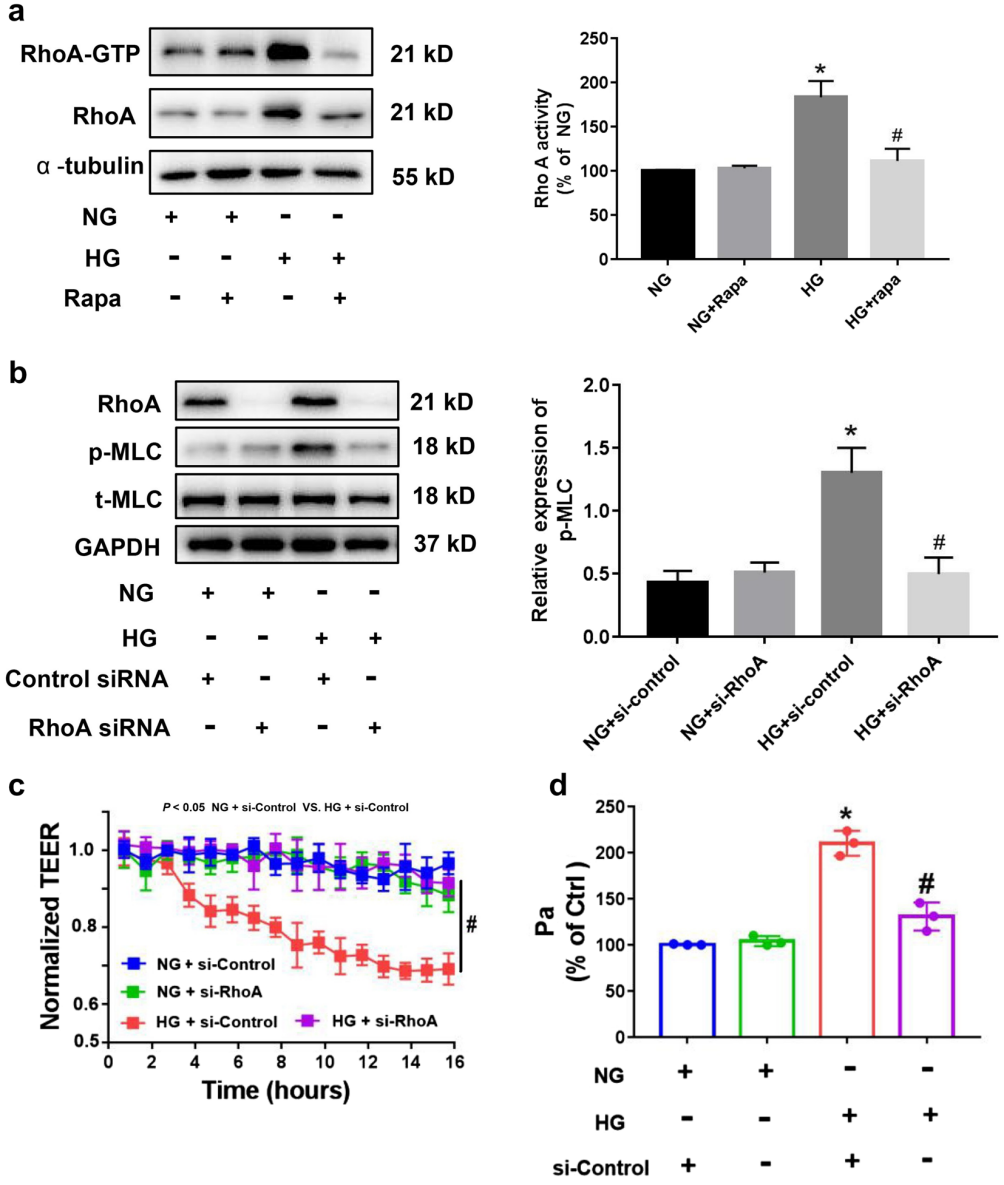
HG increased RhoA expression and activity in an mTOR-dependent manner. **a** HRGECs were treated with rapamycin for 2 h and then stimulated with HG or NG for 22 h. GTP-RhoA levels were measured via pull-down assay. RhoA expression was analyzed via immunoblotting. **b** RhoA knockdown or si-control–treated HRGECs were incubated with NG or HG for 24 h. RhoA expression and MLC phosphorylation levels were measured using western blotting. After cells were treated with si-RhoA or si-control and stimulated with HG or NG for 24 h, monolayer permeability was assessed via TEER (**c**) and HRP-albumin leakage permeability assay (**d**). HG, high glucose; HRGECs, human renal glomerular cells; NG, normal glucose; MLC, myosin light chain; TEER, transendothelial electrical resistance; HRP, horseradish peroxidase. * indicates p < 0.05 vs. the NG + si-Control group. # indicates p < 0.05 vs. the HG + si-Control group

RhoA expression and activity are critical for ROCK activation [14]. As shown in Fig. 5a, HG stimulation markedly elevated both the expression and activity of GTP-bound RhoA. Rapamycin significantly suppressed HG-induced RhoA activation and expression (Fig. 5a). These results indicated that HG stimulated endothelial cell hyperpermeability by increasing RhoA expression in an mTOR-dependent manner. Moreover, in RhoA-siRNA–treated HRGECs, HG-induced hyperpermeability and MLC phosphorylation levels were significantly downregulated (Fig. 5b). Similar to the effect on MLC phosphorylation, RhoA knockdown caused a significant increase by the TEER values and a decline in permeability (Pa) compared to those in the si-control group (*p* < 0.05) (Fig. 5c–d).

## Discussion

In this study, we confirmed that HG induced an increase in HRGEC permeability that was highly dependent on RhoA/ROCK activation and MLC phosphorylation. First, we found that HG activated the RhoA/ROCK pathway, which resulted in increased MLC phosphorylation and actin stress fiber formation, leading to endothelial hyperpermeability. Second, we observed that RhoA/ROCK pathway regulated HG-induced GFB dysfunction via mTOR activation. This pathway may be a useful therapeutic target for treating HG-induced GFB dysfunction.

GFB dysfunction is the leading cause of glomerular injury and albuminuria [2, 4]. The GFB is a composite, multilayered structure: glomerular endothelium, glomerular basement membrane, and podocytes [6, 13]. Previous studies have shown that in GFB dysfunction, glomerular endothelial cells play a key role independent of podocytes [3], and it is well established that the toxic effect of HG on the endothelial barrier eventually causes GFB [4, 9, 24]. MLC phosphorylation and F-actin stress fiber formation are crucial factors of endothelial hyperpermeability [14, 16, 23]. It has previously been reported that in human umbilical vein endothelial cells (HUVECs), increased transendothelial migration of albumin and THP1 cells occurs in response to HG, mediated by MLC phosphorylation and RhoA activation [12]. However, the underlying mechanisms remain largely unexplored.

We observed that HG exposure increased the permeability of endothelial monolayers, which is affected by MLC phosphorylation, actin stress fiber formation, and a contractile endothelial cell phenotype. The increase in permeability was highly dependent on mTOR activation and MLC phosphorylation. Inhibition of MLC phosphorylation by Y-27632 or of mTOR by rapamycin resulted in reductions in the rearrangement of the F-actin cytoskeleton and in HG-induced endothelial permeability. Our findings emphasized the significance of mTOR activation and confirmed the role of endothelial MLC-dependent F-actin rearrangement in establishing renal endothelium hyperpermeability.

Additionally, we observed upregulated RhoA expression and activity in HG-treated HRGECs. The RhoA/ROCK pathway has been shown to be involved in renal microvascular complications caused by HG conditions [25], and it has been observed that the activation of RhoA/ROCK signaling in podocytes and/or endothelial cells by HG conditions is required for the development of hyperpermeability in glomerular cells [10, 11, 17]. Consistent with these prior findings, we observed increased permeability and RhoA activity in HG-treated HRGECs. Furthermore, inhibition of RhoA/ROCK by Y-27632 attenuated MLC phosphorylation and hyperpermeability in HG-treated HRGECs. This indicates that HG-induced glomerular endothelial hyperpermeability may be mediated by the RhoA/ROCK pathway, in alignment with previous reports of RhoA/ROCK signaling in endothelial cells.

mTOR signaling has been shown to be physiologically and pathologically critical in diabetic kidney disease [19]. There is a growing body of evidence indicating that mTOR inhibitors are the preferred treatment for diabetic nephropathy [18], which is primarily associated with reduced proteinuria and albuminuria [18, 26]. Conversely in non-diabetic disease, mTOR inhibition can produce de novo proteinuria in transplant patients, which is associated with the effects of increased glomerular protein leakage induced by podocyte injury [21]. Therefore, the role of the mTOR signaling pathway in the formation of diabetic nephropathy may be cell specific, and mTOR in different cells may play different roles in the various stages of diabetic nephropathy [19, 20, 26].

Our current results demonstrated that mTOR inhibition by rapamycin suppressed HG-induced endothelial hyperpermeability in HRGECs. A recent report demonstrated that IGF-1–stimulated F-actin reorganization and cell motility occurs via the upregulation of RhoA protein expression and activity through the mTOR signaling pathway in tumor cells [22]. Similarly, our data demonstrated that RhoA expression and activity was upregulated in HG-treated cells and that this effect was partially diminished by rapamycin-mediated suppression of the mTOR pathway.

Previous studies have shown that mTOR upregulates RhoA activity in HG-treated HUVECs [12]. Here, for the first time, we demonstrated that mTOR controlled the activity and expression of RhoA in HG-treated HRGECs. This result is supported by the finding that rapamycin-mediated inhibition of mTOR inhibited the expression and activity of RhoA induced by HG conditions [19, 22]. This is consistent with previous findings suggesting that RhoA/ROCK activation and subsequent F-actin cytoskeleton contraction result in increased membrane permeability following HG exposure [11, 17, 27].

## Conclusions

In summary, our present study demonstrated that mTOR activation and the subsequent upregulation of the RhoA/ROCK/p-MLC pathway are essential for the development of HG-induced hyperpermeability in HRGECs in vitro. Thus, the inhibition of mTOR or RhoA/ROCK in glomerular endothelial cells may represent a novel therapeutic strategy for preventing hyperglycemia-induced albuminuria. mTOR-dependent RhoA expression and activity are essential for HG-induced endothelial MLC phosphorylation and F-actin cytoskeleton rearrangement. As the disruption of endothelial barrier integrity further contributes to GFB derangements, these findings provide pharmacological targets for the prevention of HG-induced GFB injury and albuminuria. These results demonstrated that HG dysregulates endothelial cell cytoskeleton rearrangement via Rho-dependent pathways controlled by mTOR activation.

## Data Availability

All data generated or analyzed during this study are included in this published Article.

## Abbreviations

HG: High glucose
HRGECs: Human renal glomerular endothelial cells
NG: Normal glucose
TEER: Transendothelial electric resistance
HRP: Horseradish peroxidase
MLC: Myosin light chain
mTOR: Mammalian target of rapamycin

## References

[1] Bonner R, Albajrami O, Hudspeth J, Upadhyay A (2020). Diabetic kidney disease. Prim Care.

[2] Satchell S (2013). The role of the glomerular endothelium in albumin handling. Nat Rev Nephrol.

[3] Jourde-Chiche N, Fakhouri F, Dou L, Bellien J, Burtey S, Frimat M, Jarrot PA, Kaplanski G, Le Quintrec M, Pernin V, Rigothier C, Sallee M, Fremeaux-Bacchi V, Guerrot D, Roumenina LT (2019). Endothelium structure and function in kidney health and disease. Nat Rev Nephrol.

[4] Ndisang JF (2018). Glomerular endothelium and its impact on glomerular filtration barrier in diabetes: are the gaps still illusive?. Curr Med Chem.

[5] Sward P, Rippe B (2012). Acute and sustained actions of hyperglycaemia on endothelial and glomerular barrier permeability. Acta Physiol (Oxf).

[6] Gil CL, Hooker E, Larrivee B (2021). Diabetic kidney disease, endothelial damage, and podocyte-endothelial crosstalk. Kidney Med.

[7] Eftekhari A, Vahed SZ, Kavetskyy T, Rameshrad M, Jafari S, Chodari L, Hosseiniyan SM, Derakhshankhah H, Ahmadian E, Ardalan M (2020). Cell junction proteins: crossing the glomerular filtration barrier in diabetic nephropathy. Int J Biol Macromol.

[8] Axelsson J, Rippe A, Rippe B (2010). Acute hyperglycemia induces rapid, reversible increases in glomerular permeability in nondiabetic rats. Am J Physiol Renal Physiol.

[9] Dou L, Jourde-Chiche N (2019). Endothelial toxicity of high glucose and its by-products in diabetic kidney disease. Toxins (Basel)..

[10] Korakas E, Ikonomidis I, Markakis K, Raptis A, Dimitriadis G, Lambadiari V (2020). The endothelial glycocalyx as a key mediator of albumin handling and the development of diabetic nephropathy. Curr Vasc Pharmacol.

[11] Wang X, Zhao X, Feng T, Jin G, Li Z (2016). Rutin prevents high glucose-induced renal glomerular endothelial hyperpermeability by inhibiting the ROS/Rhoa/ROCK signaling pathway. Planta Med.

[12] Zhao XY, Wang XF, Li L, Zhang L, Shen DL, Li DH, Jin QS, Zhang JY (2015). Effects of high glucose on human umbilical vein endothelial cell permeability and myosin light chain phosphorylation. Diabetol Metab Syndr.

[13] Ramnath RD, Satchell SC (2020). Glomerular endothelial cells: assessment of barrier properties in vitro. Methods Mol Biol.

[14] Kazakova OA, Khapchaev AY, Shirinsky VP (2020). MLCK and ROCK mutualism in endothelial barrier dysfunction. Biochimie.

[15] Pan XW, Wang MJ, Gong SS, Sun MH, Wang Y, Zhang YY, Li F, Yu BY, Kou JP (2020). YiQiFuMai Lyophilized Injection ameliorates tPA-induced hemorrhagic transformation by inhibiting cytoskeletal rearrangement associated with ROCK1 and NF-kappaB signaling pathways. J Ethnopharmacol.

[16] Xu C, Wu X, Hack BK, Bao L, Cunningham PN (2015). TNF causes changes in glomerular endothelial permeability and morphology through a Rho and myosin light chain kinase-dependent mechanism. Physiol Rep..

[17] Yin Q, Xia Y, Wang G (2016). Sinomenine alleviates high glucose-induced renal glomerular endothelial hyperpermeability by inhibiting the activation of RhoA/ROCK signaling pathway. Biochem Biophys Res Commun.

[18] Li Q, Zeng Y, Jiang Q, Wu C, Zhou J (2019). Role of mTOR signaling in the regulation of high glucose-induced podocyte injury. Exp Ther Med.

[19] Sivertsson E, Friederich-Persson M, Oberg CM, Fasching A, Hansell P, Rippe B, Palm F (2018). Inhibition of mammalian target of rapamycin decreases intrarenal oxygen availability and alters glomerular permeability. Am J Physiol Renal Physiol.

[20] Axelsson J, Rippe A, Rippe B (2015). mTOR inhibition with temsirolimus causes acute increases in glomerular permeability, but inhibits the dynamic permeability actions of puromycin aminonucleoside. Am J Physiol Renal Physiol.

[21] Puelles VG, van der Wolde JW, Wanner N, Scheppach MW, Cullen-McEwen LA, Bork T, Lindenmeyer MT, Gernhold L, Wong MN, Braun F, Cohen CD, Kett MM, Kuppe C, Kramann R, Saritas T, van Roeyen CR, Moeller MJ, Tribolet L, Rebello R, Sun YB, Li J, Muller-Newen G, Hughson MD, Hoy WE, Person F, Wiech T, Ricardo SD, Kerr PG, Denton KM, Furic L, Huber TB, Nikolic-Paterson DJ, Bertram JF (2019). mTOR-mediated podocyte hypertrophy regulates glomerular integrity in mice and humans. JCI Insight.

[22] Liu L, Luo Y, Chen L, Shen T, Xu B, Chen W, Zhou H, Han X, Huang S (2010). Rapamycin inhibits cytoskeleton reorganization and cell motility by suppressing RhoA expression and activity. J Biol Chem.

[23] Wang T, Shimizu Y, Wu X, Kelly GT, Xu X, Wang L, Qian Z, Chen Y, Garcia JGN (2017). Particulate matter disrupts human lung endothelial cell barrier integrity via Rho-dependent pathways. Pulm Circ.

[24] Fu J, Lee K, Chuang PY, Liu Z, He JC (2015). Glomerular endothelial cell injury and cross talk in diabetic kidney disease. Am J Physiol Renal Physiol.

[25] Peng H, Li Y, Wang C, Zhang J, Chen Y, Chen W, Cao J, Wang Y, Hu Z, Lou T (2016). ROCK1 induces endothelial-to-mesenchymal transition in glomeruli to aggravate albuminuria in diabetic nephropathy. Sci Rep.

[26] Wang Y, Zhang H, Pang T, Zuo Z, Ren K (2020). Rapamycin improves renal injury induced by Iodixanol in diabetic rats by deactivating the mTOR/p70S6K signaling pathway. Life Sci.

[27] Zhu L, Wang W, Xie TH, Zou J, Nie X, Wang X, Zhang MY, Wang ZY, Gu S, Zhuang M, Tan J, Shen C, Dai Y, Yang X, Yao Y, Wei TT (2020). TGR5 receptor activation attenuates diabetic retinopathy through suppression of RhoA/ROCK signaling. FASEB J.

